# An estimate of the cost of burnout on early retirement and reduction in clinical hours of practicing physicians in Canada

**DOI:** 10.1186/1472-6963-14-254

**Published:** 2014-06-13

**Authors:** Carolyn S Dewa, Philip Jacobs, Nguyen Xuan Thanh, Desmond Loong

**Affiliations:** 1 Centre for Research on Employment and Workplace Health, Centre for Addiction and Mental Health, 33 Russell Street, Toronto, Ontario M5S 2S1, Canada; 2 Department of Psychiatry, University of Toronto, 250 College Street, Toronto M5T 1R8, Canada; 3 Institute of Health Economics, Edmonton T5J 3N4, Canada; 4 Department of Medicine, University of Alberta, Edmonton T6G 2R3, Canada

**Keywords:** Cost, Burnout, Physician, Productivity

## Abstract

**Background:**

Interest in the impact of burnout on physicians has been growing because of the possible burden this may have on health care systems. The objective of this study is to estimate the cost of burnout on early retirement and reduction in clinical hours of practicing physicians in Canada.

**Methods:**

Using an economic model, the costs related to early retirement and reduction in clinical hours of physicians were compared for those who were experiencing burnout against a scenario in which they did not experience burnout. The January 2012 Canadian Medical Association Masterfile was used to determine the number of practicing physicians. Transition probabilities were estimated using 2007–2008 Canadian Physician Health Survey and 2007 National Physician Survey data. Adjustments were also applied to outcome estimates based on ratio of actual to planned retirement and reduction in clinical hours.

**Results:**

The total cost of burnout for all physicians practicing in Canada is estimated to be $213.1 million ($185.2 million due to early retirement and $27.9 million due to reduced clinical hours). Family physicians accounted for 58.8% of the burnout costs, followed by surgeons for 24.6% and other specialists for 16.6%.

**Conclusion:**

The cost of burnout associated with early retirement and reduction in clinical hours is substantial and a significant proportion of practicing physicians experience symptoms of burnout. As health systems struggle with human resource shortages and expanding waiting times, this estimate sheds light on the extent to which the burden could be potentially decreased through prevention and promotion activities to address burnout among physicians.

## Background

Globally, interest in the prevalence of burnout has grown in the healthcare professions including physicians. Conceptually, burnout is a syndrome consisting of three dimensions: emotional exhaustion, depersonalization and low personal accomplishment [1]. Estimates suggest that about one-third to one-half of physicians of various specialties experience at least one dimension of burnout (e.g., [2-6]).

The focus on burnout could partly be attributed to the increasing awareness that physicians are exposed to workplace factors putting them at risk of ongoing high work stress. Examples include long work hours [7] and work overload [8]. In turn, long-term exposure to high work stress can result in burnout [9].

Physician burnout is associated with low job satisfaction [10,11], decreased mental health [12] and decreased quality of patient care [6]. Recent evidence suggest a negative relationship between physician burnout and productivity (i.e., increased sick leave [13], intent to leave medicine [14] or change jobs [13,15]). It appears that burnout impacts both the healthcare system and the individual physician.

### Purpose

Interest in burnout and its implications for productivity is salient given the concern about healthcare labour force shortages [16]. This anxiety has extended to the physician supply [17-19]. The link between burnout and intent to leave medicine [14] suggests there are burnout costs to the healthcare system. However, few studies have explored this. To fill this gap, this study uses an economic model and results from two national physician surveys to estimate the burden of burnout associated with Canadian physician intention to leave their practice and cutback on their caseloads. Estimating the cost of burnout is an important first step to informing decisions regarding the value of implementing initiatives to decrease physician burnout.

## Methods

### The decision tree model

**Figure 1:**
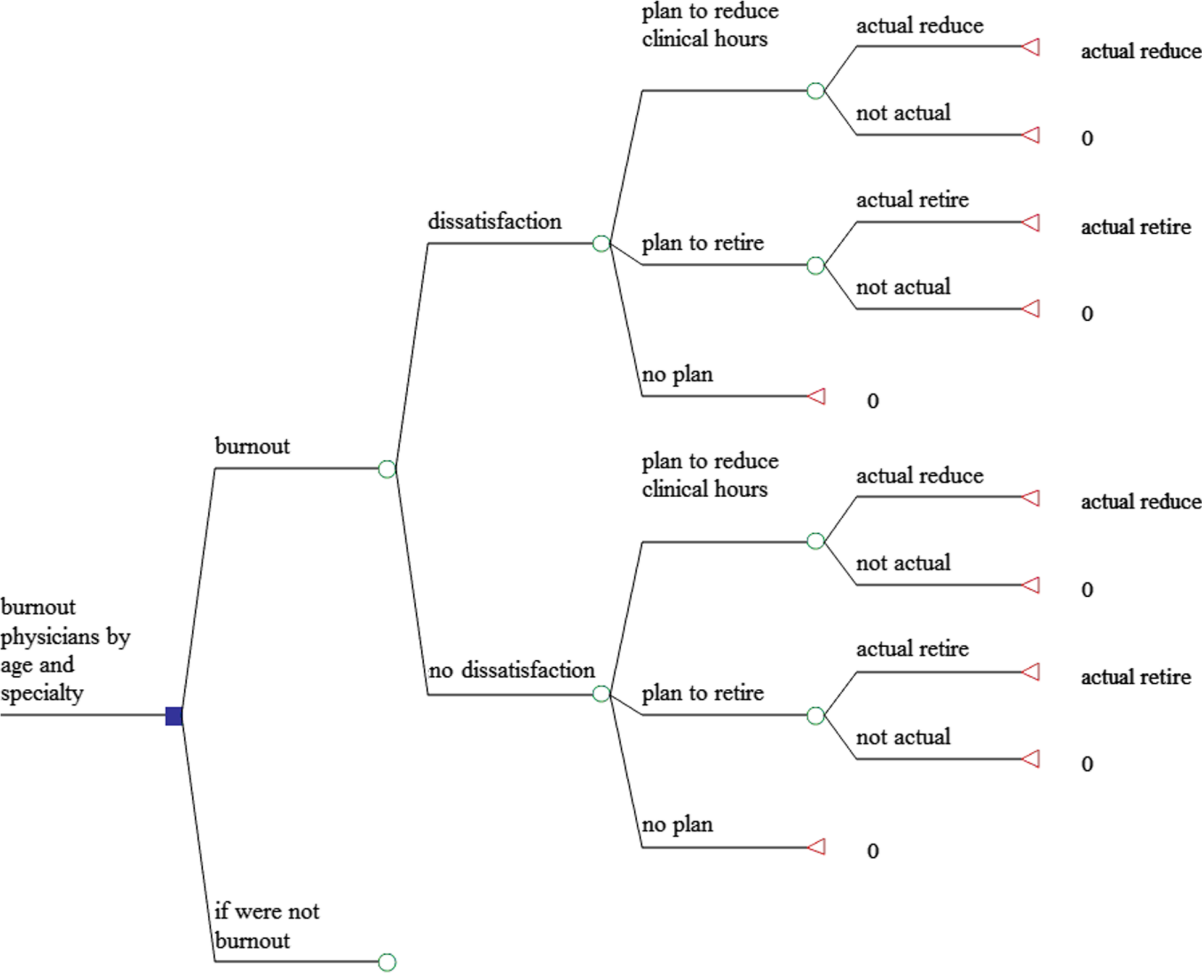
Model structure.

A decision tree (Figure 1) is used to compare the costs of two scenarios: in which physicians experienced burnout and in which those physicians did not. Both scenarios contain physicians who experience professional dissatisfaction resulting in one of three outcomes: (1) a plan to reduce clinical hours, (2) a plan to retire or (3) no plan to change work activity. Plans to reduce clinical hours or retire can result in either actual reduction or none. Figure 1 shows the pathway for physicians who experience burnout.

The pathway is based on findings from Williams et al.’s [20] study in which they looked at the relationships of stress, job satisfaction and intention to withdraw from practice among practicing physicians. They reported a significant relationship between stress and job satisfaction such that the higher the perceived stress, the lower the job satisfaction. In turn, they also found significant positive relationships between job satisfaction and intentions to leave direct patient care as well as the intention to decrease work hours. The link between satisfaction and intent to leave direct patient care has also been reported by Sibbald et al. [21] and Brett et al. [22]. In their study of physicians, Zhang and Feng [15] observed a significant inverse relationship between job satisfaction and turnover intention. The significant relationship between the burnout dimensions of emotional exhaustion and cynicism and early retirement has also been observed by Henkens and Leenders [23]. Zhang and Feng [15] found a significant relationship between turnover intention and exhaustion.

The pathway for the other scenario (those physicians who do not experience burnout) differs only with respect to the transition probabilities.

### Parameter estimates for model

The parameter estimates for the model were calculated using data from the 2012 Canadian Medical Association (CMA) Masterfile as well as datasets from two surveys of Canadian physicians. These datasets are described below.

### CMA masterfile

The January 2012 Canadian Medical Association (CMA) Masterfile was used to determine the number of physicians practicing in Canada (n = 70,700). Physicians were categorized into three major specialty groups: (1) family physicians (FPs) (n = 36,730), surgeons (n = 6,854) and other specialists (n = 25,116).

Counts were also retrieved for age groups (i.e., < 45 years, 45–54 years and 55– 64 years) by the three specialty categories (Table 1).

**Table 1:** Number of physicians in Canada by age and specialty*

**Age group**	**FP**		**Surgeons**		**Other specialists**	
	**Number**	**Mean age**	**Number**	**Mean age**	**Number**	**Mean age**
**<45 yrs**	11512	38	2822	39	7923	38
**45-54 yrs**	10868	50	2347	49	6466	50
**55-64 yrs**	9622	59	1981	59	6515	59

*Source: CMA Masterfile, Jan 2011.

### Survey data sources

Transition probabilities were calculated using two survey data sources: (1) the 2007–2008 Canadian Physician Health Survey (CPHS) and (2) the 2007 National Physician Survey (NPS).

The CPHS was conducted by the CMA, Health Canada, the Royal College of Physicians and Surgeons of Canada (RCPSC) and the Canadian Medical Foundation to collect information about the health of Canadian physicians and their health practices. The survey was sent to 8,100 randomly selected practicing physicians from the CMA membership database; 3,213 responded (41% response rate) [24].

The NPS was conducted by the CMA, College of Family Physicians of Canada (CFPC) and the RCPSC to collect information on Canadian physician practice patterns. The survey was sent to 70,000 physicians registered with either the CMA, CFPC or RCPSC, medical students and residents. The final sample was comprised of 32,026 FPs (32% response rate) and 28,785 specialists (31% response rate) [25].

### Transition probabilities

Using the three datasets described above, the parameter estimates for the model (i.e., transition probabilities) were calculated. Given the possible relationship between age and the outcomes, the transition probabilities were estimated for age and specialty groups. The following sections contain descriptions of how the estimates of the probability of burnout was calculated as well as the associated probabilities for professional dissatisfaction, retirement and work cutback outcomes.

### Burnout

The CPHS was used to calculate the proportion of physicians experiencing burnout (Table 2). The CPHS included the abbreviated Maslach Burnout Inventory-General Survey (aMBI-GS) which contains 9 of the 16 items from the full Maslach Burnout Inventory-General Survey [1,26]. The aMBI-GH has successfully been used to study burnout among physicians in the UK and Canada [27,28]. The aMBI-GH captures the three dimensions of burnout: exhaustion (EE), professional efficacy (PE), and cynicism (CYN). Each item was rated on a 7-point scale, ranging from “never” to “very often”. The mean scores were calculated for the items in each of the three sub-scales. For these analyses, three dummy variables were created to indicate whether responses for each of the sub-scales were in the highest quartile for the respective dimensions. The thresholds were compared to reported means [26,29]. For our sample, the highest quartile cut-offs were 3.71 for EE, 3.55 for CYN and 3.67 for PE. In contrast, Leiter and Schaufeli [26] reported values of 2.77 for EE, 1.75 for CYN and 4.53 for PE.

**Table 2:** Experience of professional dissatisfaction and burnout among physicians

**Experiencing**	**Dissatisfied**	
**Burnout**	**Yes**	**No**
**Yes**	35.64%	64.36%
**No**	4.52	95.48

*X*^2^ (1) = 433.08, p-value < 0.001.

Of the three burnout dimensions, the EE dimension is the most studied. The literature suggests that it is significantly related to early retirement or intent to leave [15,22,23,30]. There is evidence that CYN also has a significant association with early retirement [23]. Based on these findings, a dummy variable was created to indicate whether the respondent was in the highest quartiles for both EE and CYN. This variable was used to indicate burnout.

About 21.1% of the sample were in the top quartile for both EE and CYN. The patterns of burnout for the entire sample and the age/specialty stratified groups were not significantly different except for the FPs who were 55–64 years old; compared to the other groups, this latter group had a significantly higher prevalence of burnout (*X*^2^(2) = 20.60, p < 0.001).

### Professional dissatisfaction

The CPHS was used to calculate the transition probability from burnout to dissatisfaction. The CPHS included a single-item five-point scale to measure degree of professional satisfaction. For this analysis, the last two categories (“somewhat dissatisfied” and “very dissatisfied”) were combined to create a single category of “dissatisfied”. The remaining response categories were combined to create a category of “not dissatisfied”. For the total weighted sample, 9% of respondents reported being “somewhat dissatisfied” and 2%, “very dissatisfied”. When combined, the new category of “dissatisfied” contained 11% of the sample.

Approximately 4.5% of physicians who did not have burnout appeared to be dissatisfied while 35.6% of those who had burnout were dissatisfied professionally (Table 2). There was a statistically significant difference with regard to dissatisfaction for those who did and did not have burnout (*X*^2^(1) = 433.1, p < 0.001). Among those who had burnout, there were consistent statistically significant differences between those who were dissatisfied and those who were not. The exception was among surgeons 55–64 years old (Table 3).

**Table 3:** Experience of professional dissatisfaction and burnout among physicians by age and specialty*

**Age group**	**Specialty**	**Experiencing burnout**		**No burnout**		**Test statistic**	**P-value**
		**Dissatisfied**	**Not dissatisfied**	**Dissatisfied**	**Not dissatisfied**		
**<45 yrs**	FP	30.01%	69.99%	6.17%	93.83%	*X*^2^(1) = 41.79	<0.001
	Surgeons	43.07	56.93	4.74	95.26	*X*^2^(1) = 22.37	<0.001
	Other specialists	35.95	64.05	1.82	98.18	*X*^2^(1) = 99.87	<0.001
**45-54 yrs**	FP	37.88%	62.12%	4.74%	95.26%	*X*^2^(1) = 82.74	<0.001
	Surgeons	52.07	47.93	6.04	93.96	*X*^2^(1) = 24.43	<0.001
	Other specialists	43.05	56.95	5.01	94.99	*X*^2^(1) = 80.98	<0.001
**55-64 yrs**	FP	34.62%	65.38%	5.59%	94.41%	*X*^2^(1) = 46.15	<0.001
	Surgeons	19.90	80.10	5.71	94.29	*X*^2^(1) = 3.27	0.07
	Other specialists	25.97	74.03	3.83	96.17	*X*^2^(1) = 29.15	<0.001

*Source: CPHS 2007–2008.

### Retirement and work cutback outcomes

NPS 2007 data were used to calculate the transition probabilities from dissatisfaction/no dissatisfaction to the three outcomes. The NPS included the CPHS’ single-item five-point scale to measure degree of professional satisfaction. The dissatisfaction variable was defined in the same way as it was described above. NPS survey respondents were asked about retirement and clinic hour reduction plans for the next two years. Using the responses to these questions, the probabilities of each were calculated for those who were professionally dissatisfied and not dissatisfied.

### Plan to retire

There is a substantial difference between retirement plans for FPs who were dissatisfied versus those who were not (Table 4). For example, for FPs < 45 years, 5.3% of the dissatisfied group planned to retire within the next two years; the corresponding number for those who were not dissatisfied was 0.2%. For FPs 45–54 years, 8.4% of the dissatisfied group planned to retire; compared to 1.2% of the not dissatisfied group. For FPs 55–64 years, 24.4% were dissatisfied and planned to retire; compared to 11.0% of the not dissatisfied group. Significant differences also existed for the other two specialty groups for those 55–64 years.

**Table 4:** Physician retirement plans and experience of professional dissatisfaction by age and specialty*

**Age group**	**Specialty**	**Dissatisfied**		**Not dissatisfied**		**Test statistic**	**P-value**
		**Plan to retire**	**No plan to retire**	**Plan to retire**	**No plan to retire**		
**<45 yrs**	FP	5.32%	94.68%	0.21%	99.79%	*X*^2^(1) = 23.56	<0.001
	Surgeons	0	100	0	100	*X*^2^(1) = 0.30	0.61
	Other specialists	2.71	97.29	0.20	99.80	*X*^2^(1) = 8.57	<0.01
**45-54 yrs**	FP	8.43%	91.57%	1.21%	98.79%	*X*^2^(1) = 23.73	<0.001
	Surgeons	7.74	92.26	1.62	98.38	*X*^2^(1) = 1.31	0.22
	Other specialists	1.18	98.82	0.53	99.47	*X*^2^(1) = 0.51	0.47
**55-64 yrs**	FP	24.37%	75.63%	10.95%	89.05%	*X*^2^(1) = 11.98	<0.001
	Surgeons	44.81	55.19	13.79	86.21	*X*^2^(1) = 4.85	0.03
	Other specialists	19.58	80.42	7.10	92.90	*X*^2^(1) = 10.24	<0.01

*Source: NPS 2007.

### Plan to reduce clinic hours

When respondents were asked about plans to reduce clinic hours within the next two year, there were differences by specialty.

Among FPs, there were statistically significant differences between rates for the dissatisfied group and not dissatisfied group of FPs in all age groups (Table 5).

**Table 5:** Physician clinic hours reduction plans and experience of professional dissatisfaction by age and specialty*

**Age group**	**Specialty**	**Dissatisfied**		**Not dissatisfied**		**Test statistic**	**P-value**
		**Plan to reduce clinic hours**	**No plan to reduce clinic hours**	**Plan to reduce clinic hours**	**No plan to reduce clinic hours**		
**<45 yrs**	FP	10.69%	89.31%	5.72%	94.28%	*X*^2^(1) = 3.98	0.04
	Surgeons	0	100	0	100	*X*^2^(1) = 1.12	0.29
	Other specialists	9.17	90.83	5.72	94.28	*X*^2^(1) = 1.60	0.21
**45-54 yrs**	FP	13.46%	86.54%	7.04%	92.96%	*X*^2^(1) = 6.99	<0.01
	Surgeons	27.35	72.65	7.54	92.46	*X*^2^(1) = 4.62	0.04
	Other specialists	10.79	89.21	7.89	92.11	*X*^2^(1) = 0.93	0.33
**55-64 yrs**	FP	13.45%	86.55%	6.63%	93.37%	*X*^2^(1) = 5.65	0.02
	Surgeons	30.83	69.17	16.17	83.83	*X*^2^(1) = 1.12	0.29
	Other specialists	12.58	87.42	10.42	89.58	*X*^2^(1) = 0.29	0.60

*Source: NPS 2007.

Adjustment for planned versus actual retirement and work cutback adjustments were calculated for planned versus actual retirement and work cutback using the NPS 2007 and CMA Master File. For retirements, a ratio of actual to planned retirements for the 2-year period of the survey was calculated and indicated that 33% of those who plan to retire actually retire. For work cutbacks, similar calculations were made; it was estimated that 40% of physicians who plan to reduce working hours actually did. These adjustments were applied to all the estimates for these outcomes.

### Valuing retirement and work cutback outcomes

The most basic measures of physician services are whether the physician is practicing and the amount of time devoted to medical practice. The latter is measured by the number of hours worked during a given time period. Hours worked can be broken down into hours of regular practice, hours on-call, and hours spent doing other (non-patient) tasks such as administration and research. For physician productivity, the basic productivity measure is the number of patient visits during a given time period.

NPS 2007 data were used to estimate hours of work/week, patient visits/week and patient load/hour (Table 6). We determined the productivity losses of professional dissatisfaction on several different indicators including patients seen per work-hour, changes in work hours, and intent to retire from practice. We then attached monetary values to these to determine the impact of burnout on the healthcare system (Table 7).

**Table 6:** Physician output in Canada by specialty*

	**FP**	**Surgeons**	**Other specialists**	**Total**
**Number of physicians**	36730	6854	25116	70700
**Hours worked per week**	50.19	59.79	53.92	52.27
**Patients seen per week**	112.67	67.02	72.94	94.23
**Patients seen per hour**	2.20	1.12	1.35	1.80

*Sources: CMA Masterfile, Jan 2011; NPS 2007.

**Table 7:** Average number and cost (2010 CAD$) of patient visits per week and per year

**Specialty**	# Services per week*	# Reduced services per week*	**Cost per service†**	Cost of services per year**	Cost of reduced services per year**
**FP**	138.54	8.12 hrs	$38.47	$255,830	$15,001
**Surgeons**	107.84	21.14	80.91	418,797	82,110
**Other specialists**	114.52	4.95	62.29	342,393	14,787

†Source: CIHI 2013.

*Source: NPS 2007.

**Cost per year = services per week × 48 weeks × Cost per service.

### Hours of work

Hours were measured as total hours per week excluding on call hours. Surgeons worked the most hours (59.79 hrs/week) followed by other specialists (53.92 hrs/week) and FPs (50.19 hrs/week).

### Patient visits/week

Number of patient visits/week also were estimated. FPs saw the most patients (112.7 patients/week), followed by other specialists (72.9 patients/week) and surgeons (67.0 patients/week).

### Patient load/hour

FPs had the highest patient load/hour (2.20 patients/hr) followed by other specialists (1.35 patients/hr) and surgeons (1.12 patients/hr).

### Monetary value of services

The monetary value of services were applied according to average fee levels by specialty group, as reported in the 2010–2011 National Physician Database of the Canadian Institute for Health Information (CIHI).

### Estimating the cost of burnout

All physician data were stratified by age and specialty group. Mean ages within each group were used for the analysis. The burnout probabilities were applied to raw physician numbers and were used to estimate the number of physicians experiencing burnout. Probabilities of dissatisfaction among physicians, who were and were not experiencing burnout, were applied to the appropriate branches. Burnout cost was calculated as the difference in costs for physicians who experienced burnout versus the costs if those physicians did not experience burnout.

### Calculating cost of early retirement

Early retirement “cost” is based on the annual loss of physician revenue and is the total visits/week over 48 weeks, times the average fee/visit for the physician specialty group (Table 7). The resulting cost/year (e.g., $255,830 for FPs) is the value of services lost if the physician did not work for one year. For example, the average age of FPs in the < 45 year group was 38 years (based on CMA data for January 2012) (Table 1). These physicians had 26 years until retirement age 65. The loss in physician revenue, and hence services, would be $255,830/year, over 26 years. This figure was discounted at an annual rate of 3%.

### Calculating the cost of reduction in clinical hours

The valuation of the decrease in clinical hours was calculated by estimating the reduction in visits per week using the difference in weekly visits between dissatisfied and not dissatisfied physicians (Table 6).

Dissatisfied physicians had higher workloads; we assumed that those who planned to cut back services would reduce their services to the level of physicians who were not dissatisfied. Reductions were assumed to continue to retirement.

### Sensitivity analyses

**Figure 2:**
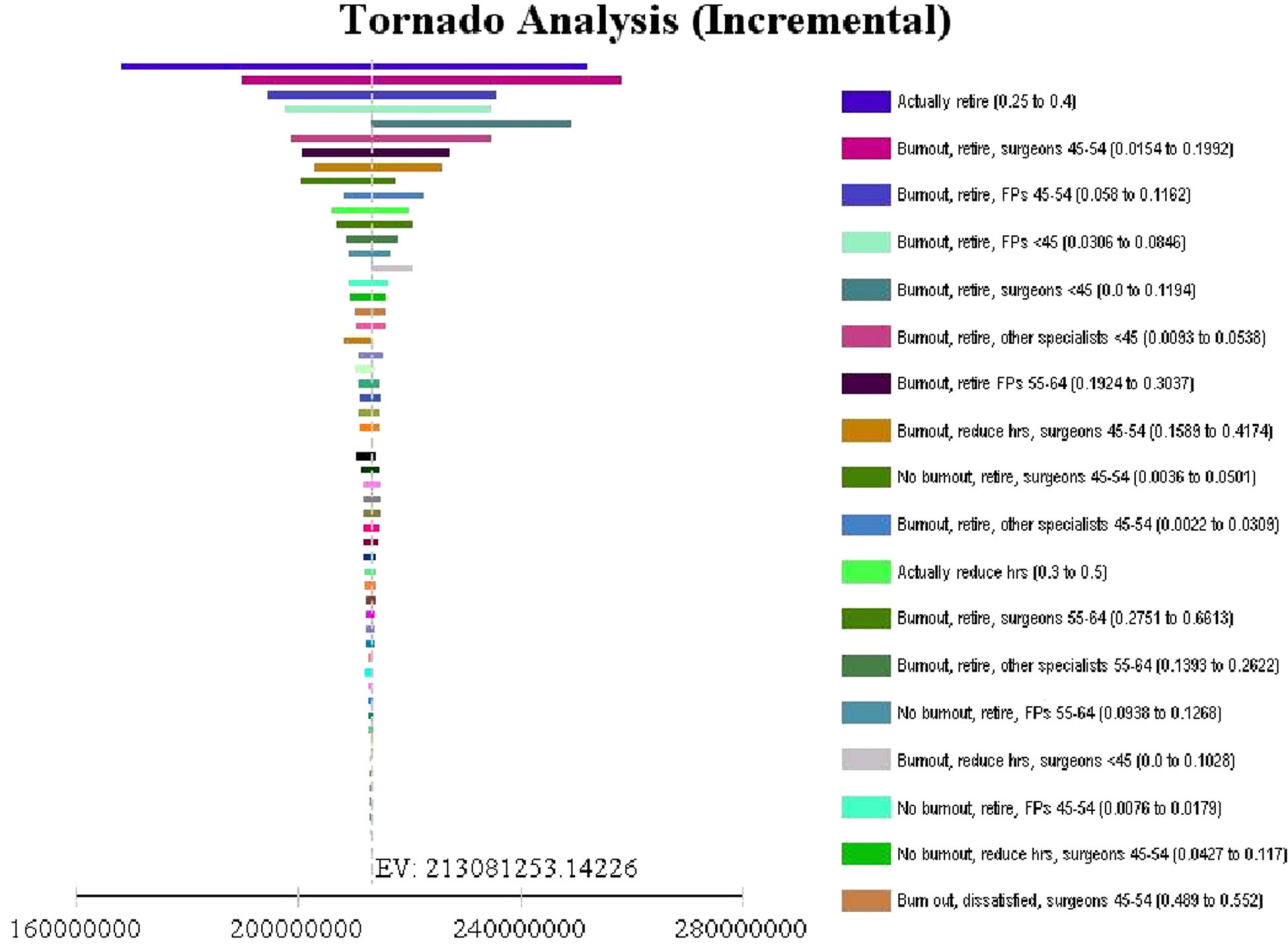
Sensitivity analysis.

Using a tornado analysis, sensitivity analyses were conducted to identify the estimates for which the cost of burnout estimate was most sensitive (Figure 2). One of the two estimates was the probability of actual retirement among those who planned to retire. For the purposes of the sensitivity analyses, the probability of actual retirement among those who planned to retire was varied from 25% to 40%. The second of the two estimates was the probability of retirement plan among surgeons who were experiencing burnout and who were 45–54 years. For the sensitivity analyses, this was varied from 1.5% to 19.9%.

## Results

### Base case

The value of the reduction in services due to early retirement and clinical hour reductions, are shown in Table 8. FP early retirements would result in service reductions totaling $118.5 million, while reduced clinical hours would result in additional $6.8 million in losses. In total, dissatisfaction, and by proxy, burnout, is estimated to result of $125.4 million service loss. Loss from reductions of surgeon services are estimated to be $52.5 million, and for other specialists, $35.3 million. The total “cost of burnout” for all physicians is estimated to be $213.1 million. This is not a one-year reduction in services. It is the future value of service reductions.

**Table 8:** Discounted cost (2010 CAD$ million) of physician burnout in Canada*

**Specialty**	**Early retirement**		**Reduced clinic hours**		**Total**	
	**$**	**%**	**$**	**%**	**$**	**%**
**FP**	$118.53	55.6%	$6.83	3.2%	$125.36	58.8%
**Surgeons**	33.68	15.8	18.77	8.8	52.45	24.6
**Other specialists**	32.96	15.5	2.31	1.1	35.27	16.6
**Total**	$185.17	86.9%	$27.91	13.1%	$213.08	100%

*Discount rate 3% annually.

Of the burnout cost, the early retirement group accounted for 86.9% and the reduced clinical load group accounted for 13.1%. FPs accounted for 58.8% of the burnout costs, surgeons for 24.6% and other specialists for 16.6%.

### Sensitivity analyses

The range in burnout costs resulting from the sensitivity analysis was from $168.2 million to $252.4 million. The results of sensitivity analyses for individual variables indicate that costs were most sensitive to the estimate for the transition probability of actual retirement among those who planned to retire. The second most sensitive transition probability estimate was for retirement plan among surgeons 45–54 years who were experiencing burnout.

## Discussion

A recent systematic literature review [31] identified five published studies from all over the world that examined the effect of burnout on physician productivity. Two outcomes with system impacts that are significantly related to burnout were intent to leave practice [14] and intent to change jobs [13,15]. In their study, Hoff and colleagues [14] found that about 44% of physicians who were experiencing burnout intended to discontinue their present practice within four years. Soler and colleagues [13] found that between 42% and 66% of physicians who experienced at least one dimension of burnout considered changing jobs. These studies highlight the potential impact of physician burnout. However, they stop short of estimating the burden of physician burnout to the healthcare system. Yet, quantifying the magnitude of the burden is critical to understanding the extent to which resources should be directed to the problem. Thus, the estimates we report in this paper are an important step towards this understanding.

Using national physician sample survey data from the CPHS 2007–2008 and the NPS 2007, we estimated the extent of physician burnout, and the impact of burnout on physician productivity. To relate burnout to productivity, we assumed professional dissatisfaction was an intermediate variable. The two types of productivity were examined: early retirement and reductions in clinical load. Our results indicated that nationally, the health service loss due to early retirement were $185.2 million and $27.9 million for reduced clinical load (Table 8). These are discounted future values in the absence of current steps to address burnout.

According to the CIHI National Health Expenditure Trends 1975–2012, Canada spent $28,924 million on physician services (public and private) in 2011. Service loss of $213.1 million is about 1% of the total physician services. The analyses use a 26 year time horizon. Thus, the estimate reflects 26 years for one cohort of physicians. In a sense, it is an aggregate value of services lost due to actions taken in a single year. Cutbacks and retirements from previous cohorts will also be felt. We did not include these in our calculation.

Our estimate is conservative because it is based on the net difference between retirement and cutbacks in services between those physicians who were very dissatisfied (adjusted by burnout proportions) and those who were not. Some of these physicians might also have cutback on services or retired, but our estimates would not have accounted for this.

In addition, Williams et al. [20] suggest that there are at least three paths leading to the intention for physicians to withdraw from practice; burnout is one of them. The other two are related to lower mental health and poor perceptions of physical health. By focusing on the pathway of burnout and dissatisfaction, we may have underestimated the effects of burnout to the extent that it contributes to poor perceptions of physical and mental health. It will be important for future studies to consider the additional costs of these other pathways.

There are several study limitations. First, we did not directly relate burnout to physician resource use. There were no data available to do this. Instead, we used modified professional dissatisfaction as a proxy for burnout. Professional dissatisfaction is highly correlated with burnout; if there were a burnout scale on the national physician survey, it would likely have shown similar patterns of resource use.

Second, because the data files could not be linked, we use intended behaviour as an approximation for actual behaviour. Our reduction adjustment factors for planned and actual service cutbacks and retirements were based on data which included both satisfied and dissatisfied physicians. If dissatisfied physicians were more likely to realize their plans than were satisfied physicians, then the ratio that we used in our base case was too low.

Third, potentially there are benefits for physicians and patients from workload reductions. Workload reductions can directly benefit physicians, in that their personal and professional satisfaction could increase. In addition, early retirements may be avoided and the quality of patient care may improve. Estimates did not account for these possibilities.

## Conclusions

A significant proportion of practicing physicians experience symptoms of burnout.

Healthcare system losses were estimated to be $185.2 million due to early retirement and $27.9 million due to reductions in clinical hours. As health systems struggle with human resource shortages and expanding waiting times, this estimate sheds light on the extent to which the burden could be potentially decreased through prevention and promotion activities to address burnout among physicians.

## Abbreviations

aMBI-GS: Abbreviated maslach Burnout inventory-general survey; CFPC: College of family physicians of Canada; CIHI: Canadian institute for health information; CMA: Canadian medical association; CPHS: Canadian physician health survey; CYN: Cynicism; EE: Emotional exhaustion; FP: Family physician; NPS: National physician survey; PE: Professional efficacy; RCPSC: Royal college of physicians and surgeons of Canada.

## Competing interests

The authors declare that they have no competing interests.

## Authors’ contributions

CSD collaborated on the design, analysis and interpretation of the data. PJ led the conception, design, data acquisition, analysis and interpretation of the data. NXT collaborated on the design, analysis and interpretation of the data. DL collaborated on the design and interpretation of the data. All authors read and approved the final manuscript.

## Pre-publication history

The pre-publication history for this paper can be accessed here:

http://www.biomedcentral.com/1472-6963/14/254/prepub
